# On the shape of the radiation survival curve in tumor spheroids: The role of oxygen heterogeneity

**DOI:** 10.1002/mp.70425

**Published:** 2026-04-03

**Authors:** Uwe Schneider, Ning Liang, Jürgen Besserer

**Affiliations:** ^1^ Department of Physics Science Faculty University of Zürich Zürich Switzerland; ^2^ Radiotherapy Hirslanden Zürich Switzerland

**Keywords:** hypoxia, oxygen diffusion, radiation cell survival, track‐event model, tumor spheroids

## Abstract

**Background:**

The shape of cell survival curves at the tissue level remains an open question in radiobiology. While homogeneous cell populations (so‐called single cells) typically exhibit an exponential decrease in survival with increasing dose, it is unclear whether this behavior persists in multicellular systems, where oxygen and nutrient gradients introduce additional complexity.

**Purpose:**

This study investigates how oxygen diffusion and consumption in tumor spheroids modify the aggregate radiation response and whether the resulting survival curves follow a purely exponential or a linear–quadratic (LQ) form on the logarithmic scale.

**Methods:**

WiDr human colon adenocarcinoma cells were modeled using the track‐event theory of cell survival. The parameters *p* = 0.04 Gy^−1^, *q* = 0.70 Gy^−1^, and *OER_max_* = 3.44 were obtained by fitting oxic and anoxic single‐cell data from West et al. Oxygen tension profiles *pO2(r)* in spheroids were calculated according to the diffusion–consumption model of *Grimes et al.* using *p_0_* = 150 Torr, *r_l_* = 216 µm, and *a* = 1.27×10^−6^ m^3^ kg s^−1^. Oxygen‐dependent, location‐specific cellular radiosensitivity was modeled via the oxygen enhancement ratio (*OER*), and the overall spheroid survival fraction was obtained by integrating survival over the viable spheroid volume, using both numerical calculation and a closed‐form analytical approximation.

**Results:**

Both the mechanistic oxygen–diffusion–based numerical survival model and the simplified approximation reproduced experimental survival data for WiDr spheroids of diameters 100–1200 µm reported by Buffa et al. and West et al. The predicted survival curves become progressively less steep as spheroid size increases, reflecting the larger hypoxic fraction. For large spheroids, both model and data exhibit a distinct *upward curvature* of the survival curve at high doses, deviating from both exponential and downward‐bending LQ behavior. This effect arises from dose‐dependent weighting of oxic and hypoxic cell subpopulations within the heterogeneous spheroid.

**Conclusions:**

The results support the hypothesis that oxygen heterogeneity fundamentally alters the apparent dose–response relationship in multicellular systems. The transition from single‐cell to spheroid‐level behavior introduces non‐linear averaging effects that produce survival curves not captured by standard exponential or LQ models. These findings provide a mechanistic bridge between cellular radiobiology and tissue‐scale dose–response modeling.

## 1 INTRODUCTION

The relationship between radiation dose and cellular survival is a fundamental concept in radiobiology, providing the quantitative basis for understanding and optimizing radiotherapy.^1^ For isolated cells in vitro, the survival fraction as a function of radiation dose has long been observed to follow an approximately exponential decrease at high doses, reflecting the stochastic nature of lethal DNA damage.^2^ Although the linear–quadratic (LQ) model is the most widely used framework in clinical radiotherapy, particularly for fractionation modeling, the present study employs the mechanistic track‐event model because it naturally reproduces the experimentally supported exponential high‐dose behavior (Appendix A). The Track‐event model assumes that radiation induces discrete, randomly distributed events leading to cell inactivation.^3^ On a logarithmic scale, this model yields a survival curve with a linear component, representing single‐event (or single‐track) lethality, and a shoulder region at low doses, corresponding to the interaction or repair of sublethal lesions. At lower doses, the linear–quadratic (LQ) model can be derived as an approximation of the track‐event model, providing a convenient empirical framework to describe cell survival.^4^, ^5^

Despite its success in reproducing clonogenic survival data from single‐cell cultures, the applicability of the track‐event model to more complex biological systems remains uncertain. In multicellular assemblies such as tumor spheroids or tissues, additional factors—most notably oxygen diffusion, nutrient gradients, and intercellular interactions—can substantially modify the spatially varying cellular radiosensitivity within the tissue.^6^ These spatial and microenvironmental heterogeneities influence both the effective dose received by individual cells and their intrinsic radiosensitivity. Consequently, the aggregate survival curve of a multicellular system may differ qualitatively from that of isolated cells, potentially exhibiting a transition from a predominantly exponential to a linear–quadratic form.^7^

A central controversy in radiobiological modeling therefore concerns whether tissue‐ or organ‐level dose–response relationships retain the purely exponential behavior expected from high‐dose single‐cell responses, or whether they display an emergent linear–quadratic dependence shaped by microenvironmental heterogeneity and resistant subpopulations.^8^, ^9^ Resolving this question is crucial for the mechanistic interpretation of radiotherapy outcomes and for extending cellular‐level models to predict clinically relevant tissue responses.

In this study, we address this question by investigating the radiation survival characteristics of tumor spheroids, which serve as a model system bridging the gap between isolated cells and organized tissues. Using a computational framework that incorporates the spatial oxygen distribution within spheroids, we simulate local variations in radiosensitivity and compute the resulting aggregate survival curves. By analyzing these curves on a logarithmic scale, we test whether the overall survival of the spheroid is best represented by a pure exponential function or by a linear–quadratic relationship. This work provides a mechanistic step toward understanding how cellular radiation responses integrate across spatially heterogeneous systems to yield organ‐level dose–response behaviour.

## 2 MATERIALS AND METHODS

### 2.1 Model overview

To investigate how spatial oxygen heterogeneity in tumor spheroids modifies the radiation dose–response relationship, an analytic model was developed that links local oxygen tension to cellular radiosensitivity and integrates the resulting survival probabilities over the spheroid volume. The goal is to determine the shape of the aggregate survival curve of a multicellular spheroid. At the single‐cell level, the radiation response is described by the track‐event model:

(1) $$S_{cell}\left(D,pO2\right)=\left(1+q\left(pO2\right)\right)\cdot D\cdot e^{-\left(p\left(pO2\right)+q\left(pO2\right)\right)\cdot D}$$

where *D* is the absorbed dose and *p(pO2)*, *q(pO2)* are oxygen‐dependent parameters.

Following a standard OER‐based scaling, we define:

(2) $$p\left(pO2\right)=\frac{p}{OER\left(pO2\right)},q\left(pO2\right)=\frac{q}{OER\left(pO2\right)}$$

where *p* and *q* are the parameters under fully oxygenated conditions (air‐equilibrated, >20% O_2_), and *OER(pO2)* is the local oxygen enhancement ratio.

This formulation ensures that both the linear (single‐track) and shoulder components of the survival curve are reduced under hypoxic conditions, consistent with experimental observations.

The overall survival of a tumor spheroid is obtained by integrating the local survival over the spheroid volume *V*, assuming homogeneous cell density:

(3) $$\begin{array}{cc} & S_{spheroid}=\frac{1}{V}\;\int_{V}S_{cell}\left(D,pO_{2}\left(V\right)\right)\cdot dV\\ & =\frac{1}{V}\int_{r_{n}}^{r_{t}}\left(1+\frac{q\cdot D}{OER\left(r\right)}\right)\cdot e^{-\frac{p+q}{OER\left(r\right)}\cdot D}\cdot 4\cdot \pi \cdot r^{2}dr \end{array}$$

where *r* is the radial coordinate, *r_t_* the spheroid radius, *r_n_* the radius of the necrotic core, and *OER(r)* is the oxygen enhancement ratio at radius *r* determined via the local oxygen pressure *pO2(r)*. Although Equation (3) is written as a spatial integration over radius, it is mathematically equivalent to integrating survival as a function of *OER*, since *OER* is evaluated at each spatial position via its dependence on the local oxygen tension *pO2(r)*. This formulation emphasizes the geometric link between oxygen distribution and overall survival

Because *OER* varies with position, the volume‐averaged survival curve *S_spheroid_(D)* generally deviates from a single exponential. This emergent behavior reflects the combined contribution of oxic, hypoxic, and anoxic regions within the spheroid. To quantify the resulting dose–response, the computed survival curves are analyzed on a logarithmic scale.

The complete modeling workflow consists of:

1. **Determining the oxygen distribution *pO2(r)*** within the spheroid by solving the steady‐state diffusion–consumption equation under spherical symmetry (Section 2.2);
2. **Determining cell survival parameters and OER scaling** (Section 2.3);
3. **Integrating local survival** over the spheroid volume to obtain the overall survival curve (Section 2.4); and
4. **Model comparison**, evaluating the shapes of the resulting curves and comparison with experimental work (Section 2.5).

This framework establishes a mechanistic link between the single‐cell track‐event behavior and the emergent survival characteristics of spatially heterogeneous tumor spheroids under varying oxygenation.

### 2.2 Oxygen distribution in tumor spheroids

Under steady‐state conditions, oxygen transport in a spheroid of radius *r_t_* is described by the diffusion–consumption equation:

(4) $$\frac{D}{\Omega \cdot r^{2}}\cdot \frac{d}{dr}\left(r^{2}\cdot \frac{dpO2}{dr}\right)-a=0,0\leq r\leq r_{t}$$

where:

- *pO2(r)*—local oxygen partial pressure (Torr),
- *D*​​—diffusion coefficient for oxygen in the spheroid (typically 2 × 10^−9^ m^2^ s^2^),
- *a*—oxygen consumption rate (m^3^ kg^−1^ s^−1^), assumed constant throughout viable regions,
- *Ω—*a constant (3.0318 × 10^7^ Torr kg m^−3^).

This linear differential equation assumes that oxygen consumption per unit viable volume is independent of *pO_2_* above a critical threshold, as supported by experimental observations.^10^ Integrating the equation under the stated boundary conditions gives the well‐known parabolic oxygen profile^11^:

(5) $$pO2\left(r\right)=p_{0}+\frac{a\cdot \Omega }{6\cdot D}\cdot \left\{r^{2}-r_{t}^{2}+2\cdot r_{n}^{3}\cdot \left(\frac{1}{r}-\frac{1}{r_{t}}\right)\right\}$$

valid in the viable region where *pO2(r)* > 0 and where *p_0_* is the surface oxygen tension which corresponds to the oxygen level in the surrounding medium or culture environment (typically ≈ 150 Torr for air‐equilibrated medium, lower if the medium is oxygen‐limited).

Following the formalism of Grimes et al.,^10^ oxygen diffusion within a spheroid is characterized by two key radii: the diffusion‐limited radius $r_{l}$ and the necrotic radius $r_{n}$.

The diffusion‐limited radius represents the maximum spheroid size for which oxygen can just reach the center (p(0)=0) and is given by

(6) $$r_{l}=\sqrt{\frac{6\cdot D\cdot p_{0}}{a\cdot \Omega }}$$

where *D* is the oxygen diffusion coefficient, *a* the volumetric oxygen consumption rate, and $p_{0}$ the oxygen partial pressure at the spheroid surface. Importantly, Equation (6) provides a direct way to estimate the consumption rate a from experimentally measurable parameters: if the diffusion‐limited radius $r_{l}$ and surface oxygen tension $p_{0}$ are known.

For spheroids larger than this critical size ($r_{t}>r_{l}$), oxygen is completely depleted at an internal radius $r_{n}$, defining the boundary of the necrotic core. To determine $r_{n}$, Grimes et al. introduce a dimensionless parameter ψ, defined through

(7) $$\psi =\frac{1}{3}\;\arccos\left(1-\frac{2r_{l}^{2}}{{r_{t}}^{2}}\right)-\frac{2}{3}\cdot \pi ,$$

which provides a compact analytical representation of the cubic diffusion equation's real root.

The corresponding necrotic radius is then expressed as

(8) $$r_{n}=r_{t}\cdot \left(\frac{1}{2}-\cos\;\psi \right).$$

This trigonometric formulation ensures numerical stability and continuity between fully oxygenated ($r_{t}\leq r_{l}$) and partially necrotic ($r_{t}>r_{l}$) spheroids. As $r_{t}$ increases beyond $r_{l}$, ψ decreases, and the ratio $r_{n}/r_{t}$ grows monotonically, reflecting the progressive expansion of the anoxic core with increasing spheroid size.

Mathematically, the introduction of ψ corresponds to expressing the single real root of the cubic steady‐state diffusion equation in trigonometric form—a common approach to ensure physical (real and bounded) solutions when solving for $r_{n}$. Geometrically, ψ parameterizes how far oxygen penetrates into the spheroid relative to its total radius, effectively encoding the balance between diffusive supply and metabolic demand.

### 2.3 Determining cell survival parameters and OER scaling

To parameterize the intrinsic radiosensitivity of the WiDr human colon adenocarcinoma cells, we used the single‐cell survival data from West et al.^11^ (Figure 2, upper left panel).

In this dataset, WiDr cells were irradiated under both oxic and anoxic conditions, providing direct information on the oxygen modification of cell survival. The data were fitted using the track‐event model of Equations (1) and (2). By fitting the oxic and anoxic data sets simultaneously, common parameters *p* and *q* (oxic) were determined together with the oxygen enhancement ratio in the anoxic situation, *OER_max_* ​, thus ensuring internal consistency between the two oxygen conditions. This approach allows a robust estimation of the radiosensitivity parameters that are directly relevant to the cellular component of the spheroid model. Uncertainties of the fitted model parameters were estimated using nonparametric bootstrap resampling. For this purpose, the original WiDr single‐cell survival dataset was resampled with replacement to generate 1000 bootstrap datasets, each containing the same number of data points as the original dataset. The track‐event model parameters p, q, and $OER_{max}$ were re‐fitted to each bootstrap sample using the same nonlinear least‐squares procedure as for the original fit. The resulting distributions of fitted parameters were used to estimate parameter uncertainties; the standard deviations of these distributions were taken as 1σ uncertainties.

To extend the model from the two experimental oxygen levels to arbitrary partial pressures, we adopted the empirical relationship proposed by Carlson et al.^12^ for V79 hamster fibroblast cells, which provides an accurate phenomenological description of the oxygen effect across a wide range of conditions. The function takes the form

(9) $$OER\left(pO2\right)=\frac{pO2_{50}+OER_{max}\cdot pO2}{pO2_{50}+pO2}$$

with parameters *OER_max_* ​ taken from the West‐fit above and $pO2_{50}$​ describing the oxygen partial pressure at which the OER is half‐maximal. While the absolute values of *OER_max_​* may differ between cell lines, the general shape of the relationship represented by *pO2_50_* has been shown to be highly conserved across mammalian cells.^12^, ^13^ Therefore, the use of the V79‐based parametrization is justified as a physiologically reasonable surrogate for the unknown WiDr‐specific dependence, ensuring a continuous and realistic interpolation between oxic and hypoxic conditions.

### 2.4 Calculation of the overall survival curve

Using the oxygen tension profiles *pO2(r)* obtained from the diffusion–consumption model (Section 2.2), local radiosensitivity parameters *p(pO2)* and *q(pO2)* were assigned to each radial shell within the spheroid according to the OER scaling described in Section 2.3.

The total spheroid survival fraction can then be determined by integrating the local cell survival *S_cell_(D,pO2(r))* over the viable spheroid volume, thus linking the spatial oxygen distribution to the overall dose–response curve by the integral of Equation (3).

Because no closed‐form analytical solution could be obtained for realistic oxygen‐dependence of *OER(pO2)*, the spheroid survival was computed numerically. The spheroid radius was discretized into concentric shells of thickness *Δr*, and the integral was approximated by a weighted sum:

(10) $$\begin{array}{cc} & S_{spheroid}\left(D\right)=\frac{1}{V}\;\sum_{r_{n}}^{r_{t}}\left(1+\frac{q\cdot D}{OER\left(r_{i}\right)}\right)\\ & \cdot e^{-\frac{p+q}{OER\left(r_{i}\right)}\cdot D}\cdot 4\cdot \pi \cdot {r_{i}}^{2}\Delta r \end{array}$$

where *r_i_* denotes the mid‐radius of each shell, *r_n_* the necrotic radius, *r_t_* the outer spheroid radius, and $V=\frac{4}{3}\cdot \pi \cdot \left(r_{t}^{3}-r_{n}^{3}\right)$ the viable spheroid volume. The discretization step *Δr* was chosen sufficiently small to ensure numerical convergence of the calculated survival fraction.

To obtain a more transparent analytical expression and to explore the dominant effect of oxygen heterogeneity, we also derived a simplified two‐region approximation. In this approach, the oxygen dependence of the OER (Equation 9) is replaced by a step approximation:

(11) $$\begin{array}{c} OER\left(pO2\right)=\left\{\begin{array}{c} OER_{max}\;\;\;\;for\;pO2\geq pO2_{50}\\ 1\;\;\;\;\;\;\;\;\;\;\;\;\;for\;pO2<pO2_{50} \end{array}\right. \end{array}$$

From the Grimes model the corresponding radius at which the pressure reaches *pO2_50_* can be calculated with:

(12) $$\begin{array}{c} r_{50}=\frac{2}{\sqrt{3}}\cdot \sqrt{r_{t}^{2}+\frac{2\cdot r_{n}^{3}}{r_{t}}+\frac{6\cdot D\cdot \left(pO2_{50}-p_{0}\right)}{\Omega \cdot a}}\\ \cdot cos\left(\frac{1}{3}\cdot arccos\left(-\sqrt{{\left(\frac{3\cdot r_{n}^{2}}{r_{t}^{2}+\frac{2\cdot r_{n}^{3}}{r_{t}}+\frac{6\cdot D\cdot \left(pO2_{50}-p_{0}\right)}{\Omega \cdot a}}\right)}^{3}}\right)\right) \end{array}$$

Cells with *r_n_ < r < r_50_* were considered anoxic (or severely hypoxic), whereas cells *r_50_ < r < r_t_* were treated as oxic. The overall survival fraction was then expressed as a volume‐weighted sum of the oxic and anoxic survival components:

(13) $$S_{spheroid}\left(D\right)=\frac{\left(r_{50}^{3}-r_{n}^{3}\right)\cdot S_{anoxic}+\left(r_{t}^{3}-r_{50}^{3}\right)\cdot S_{oxic}}{r_{t}^{3}-r_{n}^{3}}$$

This analytical approximation captures the essential influence of oxygen heterogeneity through the relative volume fractions of oxic and anoxic regions.

### 2.5 Model analysis and comparison to experiment

The calculated spheroid survival fractions *S_spheroid_(D)* were compared with experimental data for WiDr human colon adenocarcinoma spheroids of various diameters. Experimental survival data were taken from Buffa et al.,^14^ who measured spheroids with diameters of approximately 100, 500, 750, 1000, and 1200 µm, and from West et al.,^11^ who reported results for spheroids of about 1200 µm. For each spheroid size, the oxygen distribution *pO2(r)* was computed using the diffusion–consumption model described in Section 2.2, and the corresponding overall survival *S_spheroid_(D)* was evaluated numerically according to Equation (10).

Agreement between model predictions and experimental survival data was quantified using the coefficient of determination ($R^{2}$), calculated from the residuals between measured and modeled survival fractions in logarithmic survival space.

## 3 RESULTS

### 3.1 Determination of cell survival parameter and OER scaling

**FIGURE 1:**
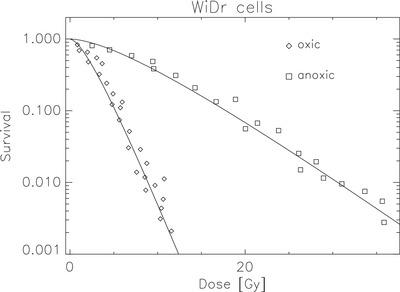
WiDr single‐cell survival under oxic and anoxic conditions. Symbols denote experimental data irradiated under oxic (◇) and anoxic (□) conditions from West et al.^11^ Solid lines show model predictions based on the fitted track‐event parameters (*p* = 0.04 Gy^−1^, *q *= 0.70 Gy^−1^, and *OERmax* = 3.44), with oxygen dependence incorporated via OER‐scaling. Separate curves correspond to oxic and anoxic conditions, respectively.

The track‐event model (Equation 1) was fitted simultaneously to the oxic and anoxic survival data of WiDr human colon adenocarcinoma cells reported by West et al.^11^ A single set of parameters *p*, *q*, and *OER_max_* was optimized to reproduce both datasets, resulting in *p *= 0.04 ± 0.03 Gy^−1^, *q* = 0.70 ± 0.04 Gy^−1^, and *OER_max_​* = 3.44 ± 0.09 (*R* ^2^ is listed in Table 1). As shown in Figure 1, the model reproduces the experimental survival curves well over the full dose range for both oxygenation conditions. The oxic curve exhibits the expected steep exponential decline, while the anoxic data show the characteristic shift toward higher survival levels due to reduced radiosensitivity. The fitted *OER_max_* value is consistent with typical experimental observations for mammalian cells, confirming the suitability of the track‐event formulation for capturing the oxygen dependence of cellular radiation response.

**TABLE 1:** Coefficient of determination ($R^{2}$) values quantifying the agreement between model predictions and experimental survival data for (i) WiDr single‐cell survival under oxic and anoxic conditions and (ii) WiDr tumor spheroid survival data for different spheroid diameters.

Type	Diameter (um)	*R* ^2^ for empirical OER model	*R* ^2^ for OER using the two region approximation
Single cell	–	0.96	
Spheroid	100	0.96	0.96
Spheroid	500	0.74	0.65
Spheroid	750	0.78	0.70
Spheroid	1000	−1.25	−1.00
Spheroid	1200	0.89	0.92

The dependence of the oxygen enhancement ratio on oxygen partial pressure was parameterized to describe the oxygen‐dependent radiosensitivity of WiDr cells.

**FIGURE 2:**
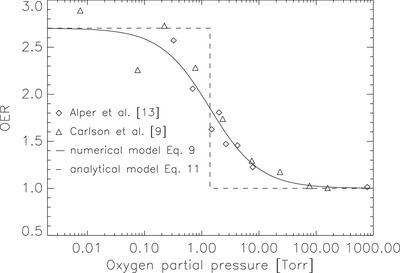
Dependence of the oxygen enhancement ratio (OER) on oxygen partial pressure $p_{O_{2}}$. Experimental data from Alper and Howard‐Flanders^13^ (diamonds) and Carlson et al.^9^ (triangles) are shown together with the fitted empirical function (Equation 9; solid line) and the simple two region approximation (Equation 13; dashed line).

Figure 2 shows the adopted *OER*–$p_{O_{2}}$ relationship used in this work. Experimental data from Alper and Howard‐Flanders^13^ (diamonds) and Carlson et al.^9^ (triangles) are displayed for comparison. The solid line represents the empirical function from Equation (9) which was fitted using $pO2_{50}=1.39$. The fitted half‐maximum pressure was subsequently used to determine the characteristic radius $r_{50}$ in the spheroid model. The dashed line represents the simple two‐region approximation from Equation (13).

### 3.2 Determination of oxygen distribution of WiDr‐spheroids

**FIGURE 3:**
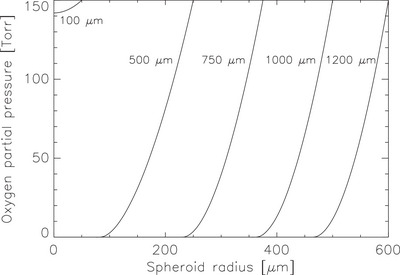
Calculated oxygen tension profiles pO2(r) for WiDr spheroids of increasing diameter, based on the diffusion—consumption model of Grimes et al.^6^ The external oxygen partial pressure was set to *p_0_* = 150 Torr, with diffusion limit *r_l _* = 216 µm and oxygen consumption rate *a* = 1.27×10^−6^ m^3^∙kg∙s^−1^. For spheroids of diameters 100, 500, 750, 1000, and 1200 µm, the corresponding necrotic radii were *r_n _* = 0, 82.3, 230, 362, and 465 µm, respectively. Oxygen tension decreases monotonically toward the spheroid center, with the development of hypoxic and ultimately anoxic (necrotic) regions in larger spheroids. These profiles provide the spatial oxygen distributions used to assign local radiosensitivity parameters for the subsequent survival curve calculations.

The spatial oxygen distribution within WiDr spheroids was calculated using the diffusion–consumption framework described in Section 2.2. For the spheroid geometry corresponding to the experimental conditions of West et al.,^11^ an external oxygen partial pressure of *p_0_* = 150 Torr was assumed, consistent with well‐aerated culture conditions. Using the experimentally observed diffusion limit radius *r_l_* = 216 µm, the oxygen consumption parameter was determined from Equation (6) as *a* = 1.27×10^−6^ m^3^∙kg∙s^−1^. This value lies within the physiological range reported for human tumor spheroids and yields realistic oxygen gradients between the spheroid surface and core. Based on this parameterization, the oxygen tension *pO2(r)* decreases monotonically from the spheroid surface toward the center, reaching hypoxic and eventually anoxic levels as diffusion limits are approached (see Figure 3). From the corresponding model Equation (8), the necrotic core radii *r_n​_* = 0, 82.3, 230, 362, 465 µm were computed for spheroid diameters 100, 500, 750, 1000, and 100 µm, respectively. In addition, the characteristic radii $r_{50}$, corresponding to the positions where $p_{O_{2}}\left(r_{50}\right)=p_{O_{2},50}$ (i.e., where the oxygen enhancement ratio reaches half its maximal value), were determined as 0, 94.8, 243, 374, and 477 µm for the same spheroid sizes. For the largest spheroids, $r_{50}$ approaches the spheroid surface, indicating that most of the viable volume is under hypoxic or anoxic conditions.

These results indicate that necrosis begins to develop for spheroids exceeding approximately 400 µm in diameter, in agreement with experimental observations for WiDr and similar human tumor spheroids. The resulting oxygen tension profiles *pO2(r)* and corresponding viable fractions were used as spatial input functions for the survival model described in Section 2.4.

### 3.3 Comparison of the modelled survival with experimental data

**FIGURE 4:**
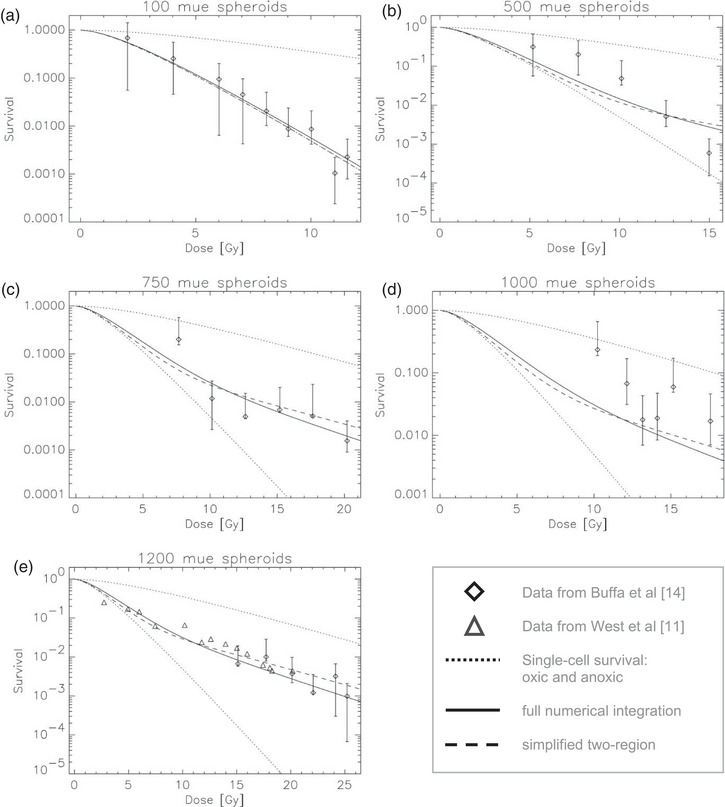
Comparison experimental and modeled survival curves for WiDr tumor spheroids of different diameters (a ≙ 100 µm; b ≙ 500 µm; c ≙ 750 µm; d ≙ 1000 µm; e ≙ 1200 µm). Symbols show experimental data from Buffa et al.^16^. Solid lines denote the full numerical integration of Equation (10), while dashed lines correspond to the simplified two‐region approximation of Equation (13). The dotted lines indicate the limiting oxic and anoxic single‐cell survival curves derived from the track‐event model.

Figure 4 compares the measured survival data of WiDr spheroids with model predictions obtained from both the full numerical integration (solid lines) and the two‐region approximation (dashed lines). For all spheroids, both models reproduce the experimental survival data well, with only small deviations at intermediate doses where the oxygen transition is most gradual.

At high doses, the two approaches converge and exhibit the same overall curvature, confirming that the observed deviations from exponential behavior primarily result from the spatial averaging of subpopulations with different oxygenation levels.

A characteristic *upward bending* of the survival curve at higher doses becomes evident for the larger spheroids. This effect arises because the anoxic core contributes disproportionately to the surviving fraction at low doses, being more radioresistant. As the dose increases, these hypoxic cells are preferentially inactivated, and the surviving population becomes increasingly dominated by the oxic outer layers, which are more radiosensitive. This dynamic shift in the relative contributions of hypoxic and oxic regions leads to an upward curvature of the aggregate survival curve on a logarithmic scale at high doses. Thus, the upward curvature does not reflect a change in the intrinsic cellular damage mechanism but rather emerges naturally from the non‐uniform oxygen distribution within the spheroid.

The good agreement between the numerical solution and the approximation confirms that this geometric and physiological weighting is the dominant factor determining the shape of the multicellular survival curve. Hence, the simplified two‐region formulation provides both a mechanistic and an analytically transparent explanation of how spatial oxygen heterogeneity gives rise to the non‐linear, upward‐bending dose–response observed in multicellular tumor systems. The coefficient of determination ($R^{2}$), for the comparison of the experimental data with the numerical and analytical models are listed in Table 1.

## 4 DISCUSSION

The presented results demonstrate that the radiation response of multicellular spheroids cannot be described adequately by a direct extrapolation of single‐cell behavior. Even when each individual cell follows a purely exponential track‐event survival relationship, spatial heterogeneity in oxygen concentration produces a qualitatively different aggregate dose–response at the spheroid level. The observed flattening of the survival curves with increasing spheroid size and the emergence of upward curvature at high doses both arise naturally from the oxygen gradients predicted by the diffusion–consumption model. These findings show that deviations from exponential or linear–quadratic (LQ) behavior in large spheroids originate not from changes in cellular repair or damage mechanisms, but from *geometric and physiological averaging*—the nonlinear summation of survival probabilities across microregions of differing oxygenation.

A particularly notable observation is the upward bending of the survival curves at high doses in large spheroids. This feature, counterintuitive within classical radiobiological models, emerges as a direct consequence of spatial heterogeneity. At low doses, survival is dominated by the more radiosensitive, oxic rim, whereas at higher doses this compartment is largely sterilized and the residual survival increasingly reflects the radioresistant hypoxic core. This dose‐dependent reweighting of subpopulation contributions leads to an upward curvature of the total survival curve on a logarithmic scale at high doses. Such behavior has also been reported in several experimental spheroid systems and may similarly contribute to the dose–response observed in macroscopic tissues and organs.

The simplified two‐region approximation provides a particularly transparent way to interpret this phenomenon. By replacing the continuous *OER*–$p_{O_{2}}$ dependence with a step function at $p_{O_{2},50}$, the spheroid is decomposed into an inner hypoxic and an outer oxic compartment, each with distinct survival probabilities. The aggregate survival then reduces to a simple volume‐weighted combination of these two contributions. Although this approach represents a coarse simplification of the continuous oxygen distribution, it reproduces the general features of the numerically integrated results very well, including the characteristic curvature and dose dependence of the survival curves. The similarity between the two solutions indicates that the essential physics of oxygen heterogeneity is governed primarily by the *fractional volumes* of oxic and hypoxic regions, rather than by the detailed functional form of the oxygen profile.

Thus, the approximation offers both a computationally efficient and mechanistically interpretable means of bridging single‐cell and multicellular survival behavior.

Although the qualitative agreement between model and data is strong, several simplifications warrant consideration. First, the model assumes steady‐state oxygen distributions and neglects dynamic processes such as diffusion transients, reoxygenation, or vascular remodeling during irradiation.

Second, the *OER*–$p_{O_{2}}$ relationship was taken from V79 cells due to the absence of WiDr‐specific measurements, which may introduce minor quantitative uncertainties. Third, the track‐event parameters p and q were derived from single‐dose survival data, not accounting for repair kinetics or dose‐rate effects. Fourth, the present model focuses exclusively on the effect of spatial oxygen heterogeneity on the shape of the spheroid survival curve. Other biological processes, such as cancer stem cell‐associated radioresistance, dose‐rate–dependent repair during irradiation, and additional sources of intrinsic radiosensitivity heterogeneity—can also influence high‐dose response and may contribute to sigmoidal or non‐monotonic survival behavior in vivo. We have not included these mechanisms here, as they would introduce multiple additional parameters and obscure the specific mechanistic role of oxygen distribution that this study aims to isolate. By starting from a single‐cell model with a well‐established exponential high‐dose limit, our analysis identifies oxygen geometry as a baseline structural factor capable of generating upward‐curving multicellular survival even in the absence of further biological complexity. Future work will extend the framework to incorporate proliferative hierarchies, stem‐cell compartments, and dynamic repair processes. Finally, necrotic regions were treated as completely non‐viable; a small fraction of metabolically quiescent but clonogenically active cells may persist near the necrotic boundary in reality. Despite these simplifications, the model provides a robust mechanistic framework that captures the dominant influence of oxygen heterogeneity on radiation survival.

It should be emphasized that the present study is performed for multicellular tumor spheroids, which constitute an experimentally well‐defined, nearly spherical model system with radially symmetric oxygen distributions. The spherical geometry and the associated diffusion–consumption description are therefore appropriate for the biological system investigated here. Nevertheless, real tumors in vivo may exhibit highly irregular shapes, heterogeneous vascular supply, and complex spatial oxygen landscapes. Extending the present framework to such geometries is certainly challenging, but it represents an essential next step toward linking the mechanistic insights obtained in spheroids to clinically relevant tumor response. Importantly, the key phenomenon demonstrated here—the emergence of distinct oxic and hypoxic compartments and their volumetric weighting leading to non‐exponential survival behavior—is expected to persist in more complex geometries. Future developments will therefore focus on image‐based oxygen fields and non‐spherical configurations to bridge the gap from spheroids to real tumors.

## 5 CONCLUSION

This study demonstrates that the radiation survival of multicellular tumor spheroids differs fundamentally from that of isolated cells, even when the same underlying track‐event model governs single‐cell response. The incorporation of realistic oxygen distributions naturally leads to survival curves that deviate from the purely exponential or linear‐quadratic form and exhibit the characteristic upward curvature observed experimentally in large spheroids. Both the full numerical integration and the simplified two‐region approximation reproduce these features, showing that the essential effect arises from the volumetric weighting of oxic and hypoxic subregions rather than from intrinsic changes in cellular radiosensitivity. The results emphasize that oxygen heterogeneity and spatial organization are key determinants of radiation response at the multicellular level. Future work should extend this framework toward dynamic oxygenation, proliferation, and vascular effects, and ultimately to tissue‐ and organ‐scale models.

Such developments would provide a quantitative bridge between cellular radiobiology and clinically relevant dose–response modeling, improving the mechanistic understanding and predictive accuracy of radiotherapy outcomes.

## CONFLICT OF INTEREST STATEMENT

The authors have no conflicts to disclose

## Figures and Tables

**FIGURE A1:**
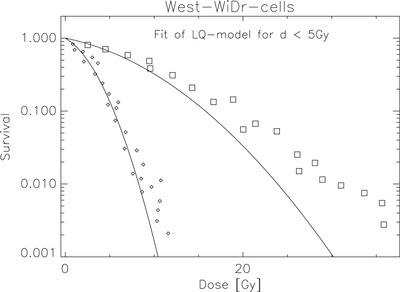
Fit of the linear–quadratic (LQ) model to WiDr single‐cell survival data for doses D < 5 Gy. Symbols represent experimental data reported by West et al. The solid curves show the model prediction based on the low‐dose fit. While the LQ model reproduces the data within the fitted dose range, it significantly deviates from the measured survival at higher doses.

**FIGURE A2:**
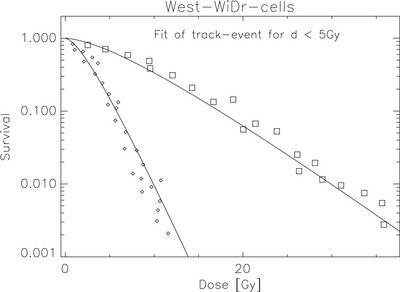
Fit of the track‐event model to WiDr single‐cell survival data for doses *D* < 5 Gy. Symbols represent experimental data reported by West et al. The solid curves show the model prediction based on the low‐dose fit. In contrast to the LQ model, the track‐event model provides a consistent description of the survival data over the full dose range.

## APPENDIX A COMPARISON OF LQ AND TRACK‐EVENT MODEL PREDICTIONS

### A.1

To clarify the choice of the track‐event model over the linear–quadratic (LQ) model for the description of single‐cell survival, we performed an additional analysis using the WiDr survival data reported by West et al. In this analysis, both models were fitted only to the low‐dose region (*D* < 5 Gy) and then extrapolated to higher doses to evaluate their predictive capability.

The results of this comparison are shown in Figures A1 and A2. In Figure A1, the LQ model was fitted to the low‐dose portion of the data. While the LQ model provides a reasonable description of the data within the fitted range, it substantially deviates from the measured survival at higher doses. In particular, the predicted survival decreases too rapidly and does not reproduce the approximately exponential behavior observed experimentally.

In contrast, Figure A2 shows the corresponding fit using the track‐event model, again restricted to doses below 5 Gy. In this case, the model extrapolates well to higher doses and continues to describe the experimentally observed survival behavior over the entire dose range. This improved predictive capability is consistent with the theoretical relationship between the two models: the LQ model can be interpreted as a low‐dose approximation of the track‐event model, whereas the latter retains the correct exponential asymptotic behavior at large doses.

These results illustrate that although the LQ model can adequately describe survival data within a limited low‐dose interval, it lacks predictive power when extrapolated to higher doses. The track‐event model therefore provides a more consistent description of the WiDr survival data across the full dose range considered in this study.
